# Morphological and Functional Characteristics of Animal Models of Myocardial Fibrosis Induced by Pressure Overload

**DOI:** 10.1155/2020/3014693

**Published:** 2020-01-31

**Authors:** Yuejia Ding, Yuan Wang, Qiujin Jia, Xiaoling Wang, Yanmin Lu, Ao Zhang, Shichao Lv, Junping Zhang

**Affiliations:** ^1^First Teaching Hospital, Tianjin University of Traditional Chinese Medicine, Tianjin 300193, China; ^2^Qian'an Hospital, Traditional Chinese Medicine, Qian'an 064400, China; ^3^Tianjin Nankai Hospital, Tianjin 300381, China; ^4^College of Global Public Health, New York University, 726 Broadway, New York, NY 10003, USA; ^5^Tianjin Key Laboratory of Traditional Research of TCM Prescription and Syndrome, Tianjin 300193, China

## Abstract

Myocardial fibrosis is characterized by excessive deposition of myocardial interstitial collagen, abnormal distribution, and excessive proliferation of fibroblasts. According to the researches in recent years, myocardial fibrosis, as the pathological basis of various cardiovascular diseases, has been proven to be a core determinant in ventricular remodeling. Pressure load is one of the causes of myocardial fibrosis. In experimental models of pressure-overload-induced myocardial fibrosis, significant increase in left ventricular parameters such as interventricular septal thickness and left ventricular posterior wall thickness and the decrease of ejection fraction are some of the manifestations of cardiac damage. These morphological and functional changes have a serious impact on the maintenance of physiological functions. Therefore, establishing a suitable myocardial fibrosis model is the basis of its pathogenesis research. This paper will discuss the methods of establishing myocardial fibrosis model and compare the advantages and disadvantages of the models in order to provide a strong basis for establishing a myocardial fibrosis model.

## 1. Introduction

Myocardial fibrosis is a pathological process characterized by cardiomyocyte injury, alterations of the cardiac extracellular matrix, and dysregulated collagen turnover [1]. As a pathological basis for a variety of heart diseases, it is a potential cause of sudden cardiac death [2]. It usually involves various mechanisms, such as oxidative stress, the renin-angiotensin-aldosterone system (RASS), inflammatory factors, cytokines, vasoactive substances, and signal transduction pathways. More importantly, pressure overload, as a common predisposing factor of cardiac remodeling, plays a critical role in the pathogenesis of fibrotic cardiomyopathy [3]. Therefore, selection of a suitable pressure-load-induced myocardial fibrosis model is the primary condition for studying its pathological features, pathogenesis, and treatment. In this paper, the excellent modeling methods are summarized, with the hope of providing a reference for researchers.

## 2. Determination of Myocardial Fibrosis Animal Models

Primates, as the closest relatives to humans, seem to be a suitable choice for animal models establishment, but these models are expensive and technically complex; therefore they are rarely used in experimental research. Much of our understanding of the complex mechanisms of myocardial fibrosis has come from experimental studies in other kinds of animals, such as dogs, pig, sheep, and rodents [1]. The reproductive cost of rodents is much lower than that of large animals, which increases the number of animals in the study and improves the statistical ability [4]. So rodents tend to be the mainstream of experimental animals. These animal models can be produced via different techniques, mainly surgery, pharmacology, and gene manipulation.

## 3. Methods and Evaluation of Establishing Animal Models

### 3.1. Spontaneous Hypertension Model

Spontaneously hypertensive rat (SHR) is a well model of hereditary hypertension and hypertensive cardiomyopathy [5]. The most commonly used experimental animal is a Wistar inbred rat cultivated by Okamoto in 1963. The spontaneous hypertension in this model is high, which is closely related to the activation of renin-angiotensin-aldosterone system (RASS) [6].

Generally 4 weeks after birth in rats, blood pressure will be significantly increased and left ventricular hypertrophy will occur, which was characterized by an increased left ventricular mass/weight body [7]. At 10 weeks, myocardial collagen content increased significantly [8]. Over time, the rat's heart contraction function gradually decreased, and diastolic dysfunction occurred 3 months later [7]. With the increase of cardiomyocyte hypertrophy and myocardial fibrosis, cardiomyocyte changes from stable hypertrophy to decompensation [5, 9, 10]. At about 18 months of age, SHR begin to show signs of heart failure, and by 24 months of age, more than 50% of rats have developed heart failure [9].

SHR model develops into hypertension and myocardial fibrosis under natural conditions without any artificial intervention and its progression of cardiac remodeling to heart failure is similar to that of humans [11]. Thus, they are more inclined to study genetic determinants and pathophysiological changes in disease progression [12]. However, the utility of the SHR in studying human hypertensive heart disease has been questioned because a genetic locus in SHR affects LV mass independent of blood pressure [13].

### 3.2. Aortic Stenosis Model

#### 3.2.1. Transverse Aortic Constriction Model (TAC)

Transverse aortic constriction model mainly elevates the afterload by ligating the aortic arch, then resulting in left ventricular hypertrophy and tissue remodeling, which is characterized by increased diameter of myocardial myocytes, accumulation of intercellular collagen, and left ventricular function impairment, ultimately leading to heart failure and death [14]. At the molecular level, the increase of blood pressure is related to Ang II AT_1_ receptor activation [15].

At present, there are different opinions about the establishment of this model. Some researchers confirmed the development of myocardial fibrosis in rats could be achieved by ligating the transverse aorta with 18-gauge needle and 4-0 silk suture for 8 weeks [15]. Its mortality rate and the success rate of the model were 7.14% and 85.71%, respectively [15]. Also myocardial fibrosis can be achieved with 17-gauge needle and 6-0 silk suture at the same observation time [16]. Other studies have shown that cardiac hypertrophy, fibrosis, and dysfunction could also be observed 4 weeks after ligating the rat aortic arch with 27-gauge needle and 7-0 silk suture ligature, presenting as an increase in heart weight index (HW/BW), cross-sectional area of left ventricular myocytes, and interstitial collagen content and a decrease in left ventricular eject fraction [17–19]. On the contrary, some studies used needle and silk sutures of the same gauge to observe significant hypertrophy and fibrosis on day 7 after TAC [20, 21]. Suture-based TAC using a 27-G needle results in varying mortality rates, that is, <25% [22–25], 25–50% [26–28], and 50–75% [29–31], and about 28% of TAC mice develop HF, accompanied by more myocardial fibrosis [32]. The same result can also be obtained with 20-gauge needle and 3-0 silk suture for 6 weeks, and in the 14-week experiment, the mortality in the TAC/Sham group was about 85% [33]. Zhao et al. used 22-gauge needle and 4-0 silk suture to ligate the aortic arch for 2 months and also obtained the pathological results of myocardial fibrosis [34]. After ligating the artery with 18-gauge needle and 5–0 nylon suture for 8 weeks, collagen deposition appeared in LV apex, accompanied by changes in fibrotic and extracellular remodeling markers [35]. From the 33 rats that underwent TAC surgery, 14 of the rats developed heart failure and 11 developed left ventricular hypertrophy, and the model success rate was 75.76% [35]. Nowadays, more and more people use titanium clips to ligate the transverse aorta to build the model. The titanium clamp was placed on the aortic arch to contract the internal diameter to 0.45 mm, and left ventricular hypertrophy occurred 3 weeks later, accompanied by mild fibrotic remodeling [36]. Echocardiography showed that all the surgical animals showed the required pressure gradient at the stenosis during the induction of the model, with a mortality rate of 20% [36]. With rat transverse aorta ligated with titanium clip with a diameter calibrated to 26-G needle, an acute myocardial small-fibre fibrosis was increased after 3 days, the network of small collagen fibres was enlarged after 2 weeks, and obvious myocardial fibrosis was observed after 4 weeks [37]. Besides, cardiac systolic dysfunction and myocardial fibrosis could also be observed after 4 weeks of transverse aortic ligation with a 30-G titanium clip [38, 39]. Research shows that the damage of the heart depends on the degree of constriction of the aortic arch [29, 40], and younger mice may take longer to develop dilated cardiomyopathy than older mice after TAC surgery [41].

Quantifying the pressure gradient across the aortic stenosis and then stratifying the left ventricular hypertrophy are the greatest advantages of this model [4], but the early mortality rate of rats is high (about 30%), which is believed to be related to acute cardiac insufficiency [42].

#### 3.2.2. Ascending Aorta Constriction Model

The ascending aortic banding is one of the common models for creating pressure overload left ventricular hypertrophy and heart failure [43]. After the increase of cardiac afterload, a variety of molecular and cellular pathways are activated, leading to cardiac remodeling through structural and functional alterations [44, 45].

The modeling method of the model is similar to the coarctation of thoracic aorta, mainly through sutures or application of metallic clips to narrow the ascending aorta. Six weeks after the ascending aorta was sutured with a 0.8 mm blunt steel wire and a 3-0 silk suture, signs of left ventricular hypertrophy with a concentric configuration and myocardial fibrosis appeared [46]. In this modeling method, mortality during model induction was approximately 7.7%, and the success rate of the model is about 82.82% [46]. Myocardial fibrosis could also be produced by surgery with a 7–0 silk suture and a 27-gauge needle for 8 weeks, and the survival rate was 30% in the untreated surgery group [47]. Similarly, a titanium clip was used to shrink the ascending aorta to 50%–60% of its original diameter, left ventricular hypertrophy and elevated pressure gradient were observed throughout echo studies in rats after 8 weeks [43], and myocardial fibrosis was observed at 21 months [48]. The mortality rate of the animals in this operation was only 20%, and there were no postoperative complications [43]. Recently, a new method of constricting the ascending aorta in mice with a fixed-diameter O-ring was studied. Using O-rings with an ID of 0.61 mm or 0.66 mm, fibrosis remodeling was present at 2 weeks or 4 weeks after surgery [49]. The postoperative survival rate of ORAB was 98.7%, and only 2 of 152 cases died in the first 2 weeks after surgery [49].

Each modeling method has its own advantages; inserting the clip around the aorta makes the surgical procedures less complicated and less time-consuming than using sutures [43]. The main advantages of ORAB are high intra- and intersurgeon reproducibility, low postoperative mortality, and reproducible HF phenotypes [49].

#### 3.2.3. Abdominal Aortic Coarctation Model (AAC)

Abdominal aortic coarctation leads to changes in hemodynamics and continuous increase in cardiac afterload, and, over time, compensatory myocardial hypertrophy gradually develops into pathological hypertrophy characterized by myocardial fibrosis [50]. The pathological process of myocardial fibrosis in this model is related to the activation of RAAS system and expression of NF-*κ*B/TGF-*β*/Smad_2_ signaling pathway [51].

The study showed that the left ventricular hypertrophy occurred after the abdominal aorta was ligated together with a 22-gauge needle and a 2-0 silk suture for 3 weeks, and myocardial fibrosis was observed at 12 weeks [52]. In addition, a recent study showed that, with abdominal aorta ligated with a 4-0 line and a 24-G needle, rats developed compensatory central hypertrophy at 4 weeks and decompensated at 12 weeks, which was characterized by the destruction of myofibrils and disorganized arrays of sarcomeres [53]. What is more, with the 2-0 line and 24-G probe ligating the abdominal aorta between the branches of the coeliac and anterior mesenteric arteries for 3 weeks, rats developed significant interstitial and perivascular fibrosis with a lower acute mortality rate (8%) [54]. The time point of development of myocardial fibrosis in rats seems to be related to the size of the needle. Using the 4–0 line, rats with a 7-G needle to narrow the abdominal aorta developed myocardial fibrosis at 4 weeks with a mortality rate of 18.8% [55], while those using a 21-G needle took only 2 weeks (mortality unknown) [56].

Abdominal aortic coarctation model is mainly used to study cardiovascular damage in hypertension [57]. In this model, the blood pressure gradually increases and the fluctuation range is small [52].

### 3.3. Renovascular Model

After renal ischemia, the renin-angiotensin system is activated, and Ang II through stimulation of AT1 receptor causes vasoconstriction, endothelial dysfunction, thrombosis, inflammation, and fibrosis [58].

#### 3.3.1. Two-Kidney One-Clip Model (2K1C)

Research showed that after the right renal artery was bluntly separated and a polytetrafluoroethylene tubing (0.2 mm internal space) was placed on the renal artery to create partial occlusion, myocardial remodeling, characterized by myocardial fibrosis, was evident at day 3 following surgery, and overall mortality and renal atrophy rate for the wild type with surgery were 1.2% and 73.3 ± 15.4% [59]. In another study that used a stainless steel wire (diameter: 0.3 mm), the occurrence time of fibres was at 21 days, and underperfusion of the right kidney was successful in all animals [60]. Placing a silver clip (0.2 mm internal space) on the renal artery to create partial occlusion, the blood pressure in the rats increased significantly 7 days after surgery [61]. At 4 weeks, obvious myocardial fibrosis was observed, which was consistent with the increased markers of fibrosis (collagen high I and collagen high III and fibronectin) [61]. In 3 other studies that used needles and thread of the same specification, the occurrence time of myocardial fibrosis was different at 15 days [62], 6 weeks [63], and 17 weeks [64].

In the 2K1C model, hypertension is maintained by the activated renin-angiotensin system, and the model is used to demonstrate the pathophysiology of a variety of transgenic lines; also researchers should pay attention to renal infarction caused by clips [65].

#### 3.3.2. Two-Kidney Two-Clip Model (2K2C)

The method of establishing two-kidney two-clip model is similar to that of two-kidney one-clip model. One silver clip with an internal diameter of 0.30 mm was placed around the left and right renal artery, respectively, for partial occlusion, blood pressure was increased at 4 weeks after the operation, and cardiac hypertrophy occurred at 10 weeks, which was characterized by increased left ventricular weight index and diffuse interstitial and perivascular fibrosis [66, 67]. Besides, Li et al. observed cardiac fibrosis by Masson's trichrome staining 4 weeks after surgery [68]. In addition to the occurrence of myocardial fibrosis, all experiments triggered cardiac dysfunction.

The hypertension induced by the two-kidney two-clip model was stable and sustained [67]. In comparison with the two-kidney one-clip model, the experimental animals with the two-kidney two-clip model are more prone to stroke and have a higher mortality rate [69].

#### 3.3.3. One-Kidney One-Clip Model (1K1C)

Unilateral nephrectomy was performed on the experimental animals and the renal artery was subsequently clipped with a sliver clip (0.15 mm internal gap); hypertension with cardiac hypertrophy and myocardial fibrosis was detected 4 weeks after the operation [70, 71].

1K1C model is often used in the study of hypertension, and the blood pressure in 1K1C model increased faster and was higher than that in 2K1C model [65]. In addition, the 1K1C model appears to be relevant for evaluating the effects of sympathoinhibitory drugs on hypertensive heart disease [72].

### 3.4. Unilateral Nephrectomy Model

Kidney disease was associated with high incidence of cardiovascular complications [73]. Nephrectomy causes water and sodium excretion disorders, resulting in increased blood volume, extracellular fluid volume, ventricular pressure, and volume overload; this ultimately leads to cardiac disease. Activation of RASS system [74] and parasympathetic disorder [75] are considered to be the pathogenic factors of nephropathy and its cardiovascular complications.

The 5/6 nephrectomy model is considered a classic model for studying cardiovascular complications of kidney disease. Recent studies have shown that cardiac hypertrophy, impaired cardiac function, and increased myocardial fibrosis occurred 8 weeks after nephrectomy in mice [73, 76]. In experimental CKD mice, myocardial fibrosis occurred 4 weeks after nephrectomy with lower mortality rate (about 20%) [77]. However, in other experiments, myocardial fibrosis on histology was first detected at 5 weeks (mortality unknown) [78]. Similarly, myocardial fibrosis can also be induced after 10 days of right nephrectomy, which is consistent with an increase in collagen content [74]. But Chang et al. observed only impaired cardiac relaxation without myocardial fibrosis 8 weeks after left nephrectomy [79].

Renal function impairment model is an excellent model for evaluating the effects of renal failure on the heart and is characterized by renal dysfunction and heart damage [80]. It is worth noting that damaged ureter and infection will increase the risk of death in experimental animals [77].

### 3.5. Pulmonary Hypertension Model

Pulmonary hypertension is characterized by increased pulmonary vascular resistance, resulting in increased right ventricular load, which eventually leads to right ventricular hypertrophy, or even heart failure and death [81]. Several mechanisms about the mechanism of induced ventricular remodeling have been put forward, including the activation of humoral factors, oxidative stress, metabolic, autophagy, apoptosis, and mitochondrial dysfunction [82]. The main models are pulmonary artery banding model, monocrotaline model, and SuHx model.

#### 3.5.1. Pulmonary Artery Banding Model

Pulmonary artery banding (PAB) is usually performed by placing a suture or clip around the pulmonary trunk proximally to the RV. Research shows that a surgical hemoclip placed around the pulmonary artery left it constricted to a diameter of 0.35 mm; three weeks later, rats developed progressive RV hypertrophy accompanied by fibrosis [83]. However, Luitel et al., using the titanium clip to contract the pulmonary artery to the same extent, observed that the mRNA expression levels of hypertrophic and profibrotic markers were significantly increased in RV tissues 3 days after PAB, with obvious myocardial fibrosis observed after 7 days [84]. Another research confirmed that a 7-0 prolene suture and an 18-gauge needle were placed around the pulmonary artery, leaving it constricted to the diameter of the needle; four weeks later, rats developed progressive RV hypertrophy accompanied by fibrosis with the mortality rate of 35.71% after surgery [85]. Besides, Eva Amalie Nielsen et al. used adjustable vascular cuff (5 mm width) to contract the pulmonary artery, and extensive RV and LV fibrosis were observed 3 weeks after PAB; during the experiment, only one experimental animal was sacrificed due to wound infection (mortality rate: 3.8%) [86]. An experiment has shown that the production of myocardial fibrosis is related to the degree of pulmonary artery contraction and the magnitude of the pressure load. A 7-0 prolene suture was used to bypass the PA and it was tied together with a 16-G or 18-G needle; 21 days later, animals with a 18-gauge needle showed decompensation of right ventricular hypertrophy, manifested as systolic-diastolic dysfunction, right heart hypertrophy, and interstitial fibrosis, while mild pulmonary retraction only resulted in compensatory hypertrophy and preserved function [87]. Using a semiclosed clip with an inner size almost equal to outer size of 18-G needle to clamp the pulmonary artery, at 4 weeks, the percentage of fibrosis in rats was significantly increased in the experimental group, and the mortality was 46.43% between weeks 4 and 8 after surgery [85].

Numerous studies have shown severe heart damage caused by PAB. However, it may have some important shortcomings such as high operative mortality due to bleeding, cardiac arrest, or pulmonary thrombosis [85].

#### 3.5.2. Monocrotaline Model

Monocrotaline (MCT) is a pyrrolizidine alkaloid extracted from the seeds of the leguminous plant [88]. Its pulmonary toxicity usually leads to pulmonary vascular disease [89]. Because the hepatic metabolism and animal response to MCT are different, rats became the mainstream choice for experimental animals with MCT-induced PH [90].

Now, MCT is widely used to induce pulmonary hypertension in rodents. The standard dose for establishing the model is 60 mg/kg body weight [91–94]. With MCT (60 mg/kg) application for 14 days, rats developed right ventricular hypertrophy, pulmonary dysfunction, and remodeling, expressed as increased weight ratio of right ventricle to left ventricle, cross-sectional area of cardiomyocytes, and PA hyperplasia [94]. Another study showed that animals developed RV hypertrophy and pulmonary dysfunction at 2 weeks after MCT administration, and significant fibrotic deposition was observed at 3 weeks [93]. Similarly, after feeding the experimental animals with MCT (60 mg/kg) for 4 weeks, the regional myocardial dysfunction and fibrosis increased, and 31.58% [86] or 37.5% [95] of PAH rats died during the 4-week experimental period. Cardiac hypertrophy and fibrosis also could be detected at 35 days and the survival rate of experimental animals was reduced by 55% [96]. Bruce et al.'s experiments showed that animals also presented right ventricular remodeling and myocardial fibrosis after 50 mg/kg of MCT treatment for 2 weeks [97]. This may be related to different animal species. However, the high doses of MCT may not necessarily induce myocardial fibrosis, and the severity of RV dysfunction is closely related to the initial dose. After 4 weeks of low-dose (30 mg/kg) and high-dose (80 mg/kg) MCT, heart failure was only observed in the high-dose group, but under microscopic observation, no interstitial fibrosis or replacement fibrosis was found in MCT group, nor were there any differences in perivascular fibrosis [90].

This model is widely used and has been proven to be efficient and reproducible [98, 99]. MCT model continues to influence preclinical PAH studies and is used to test new drugs [100]. But a study has shown that the myocardial toxicity of MCT is the leading cause of right heart failure, not pulmonary hypertension [101].

#### 3.5.3. SU5416 Model

As an inhibitor of vascular endothelial growth factor (VEGF) receptor, SU5416, together with hypoxia, can lead to the occurrence of PAH in rats [102]. Taraseviciene-Stewart et al. demonstrated for the first time that SU5416 combined with chronic hypoxia could result in severe pulmonary hypertension and pulmonary vascular remodeling [103]. Most researchers reported changes in pulmonary blood vessels and myocardium at 3 weeks of SU5416 injection (20 mg/kg) and 10% O_2_ exposure [104–107]. One study showed that early fibrosis was first detected at 5 weeks (exposure to hypoxic state for 3 weeks and normoxic state for 2 weeks) in SU5416 rats, and, at the cellular level, the signs of cardiomyocyte degeneration appeared, along with various degrees of collagen deposition [105]. Throughout this study, one of the eight rats died at 5 weeks after the SU5416 administration [105]. In another experiment, almost 25% of rats died during the first 5 weeks of SU5416 injection by using the same experimental method [108]. Other studies have shown that the same results can be seen at 8 and 9 weeks after hypoxia treatment [106, 107].

This model can be used to investigate endogenous mechanisms of the repair/reversal of pulmonary arteries and right ventricular remodeling, as well as the development of new therapeutic drugs [109].

### 3.6. Exogenous Induction Model

#### 3.6.1. NaCl

By activating the RASS system, high salt intake enhances the expression of angiotensin II receptor, increases the cardiac afterload, and accelerates cardiac interstitial fibrosis and perivascular fibrosis in the early stage of hypertension [110].

Salt-sensitive rats are commonly used to build such models, by which the time nodes of myocardial fibrosis observed are different. Feeding rats with 8% NaCl for 5 weeks could result in hypertension and compensatory left ventricular hypertrophy [111], and rats developed myocardial fibrosis along with increased collagen at 13 weeks with mortality of 68% [112]. In addition, other experiments have shown that the pathological manifestations of myocardial fibrosis could be observed at 4 weeks [113], 6 weeks [114], 8 weeks [115], 14 weeks (mortality rate: 67.0% [116] or 25% [117]), and 18 weeks (mortality rate: 25.0%) [118] after rats were fed with the same 8% NaCl.

Salt-sensitive rats are commonly used to study salt-sensitive hypertension and renal end-organ and cardiac damage [116]. But one study showed that salt-induced cardiac hypertrophy and fibrosis were not associated with blood pressure [119].

#### 3.6.2. Angiotensin II

Angiotensin II (Ang II), a central active component of RASS, mainly promotes the proliferation and differentiation of cardiac fibroblasts (CFBs) by activating TGF-*β* and MAPKs pathways and increases the synthesis of extracellular matrix (ECM) to form myocardial fibrosis [120].

At present, angiotensin is widely used to construct myocardial fibrosis model, but the dosage and time point have not reached a unified standard. Research showed that myocardial fibrosis could be induced by continuous infusion of angiotensin at different rates of 0.2 or 2.0 *μ*g/kg/min (no mice died during the 4 weeks of Ang II infusion) [121], 200 ng/kg/min [122], 1.5 *μ*g/day [123] and 0.83 *μ*g/kg/min [124], 400 ng/kg/min [125], and 0.7 mg/kg/d [126] via an osmotic minipump for 4 weeks. In addition, myocardial fibrosis could also be induced by continuous infusion of angiotensin at the rates of 1.5 mg/kg/day [127], 1000 ng/kg/min [128], 1.1 mg/kg/day [129],and 800 ng/min/kg [130] via an osmotic minipump for 2 weeks. These differences may be related to different types of animals and equipment used in experiments.

In existing mouse models of hypertension, the infusion of Ang II is widely used because it improves BP in a reliable way [131]. Because of the human relevance of the renin-angiotensin system, this model is often used to study the mechanism of angiotensin-induced hypertension on terminal organ damage [132, 133].

#### 3.6.3. Aldosterone

It is well known that excessive secretion of aldosterone (ALD) causes water and sodium retention, increases blood volume, and may ultimately contribute to cardiac hypertrophy and fibrosis. The binding of aldosterone with related receptors increases the level of reactive oxygen species in cardiac fibroblasts, induces type I and type III collagen expression and fibroblast proliferation, and promotes the formation of myocardial fibrosis [134]. Cardiac fibrosis models induced by aldosterone can be divided into two types: aldosterone alone and aldosterone plus unilateral nephrectomy.

*(1) Aldosterone Alone*. Hypertension and cardiac damage were caused by the combination of excess aldosterone and salt. Most researchers added 1% NaCl to mouse drinking water, the solvent-soluble ALD osmotic micropump was implanted subcutaneously in rats and infused at the rate of 0.2 mg/kg/day for 4 weeks, myocardial cell cross-sectional area increased by 35%, and BNP, a marker of myocardial hypertrophy, increased significantly and myocardial fibrosis appeared [135]. Another study showed that infusing aldosterone continuously at 200 mg/kg/day by subcutaneously implanted osmotic minipumps for 4 weeks also induced myocardial fibrosis [136].

*(2) Aldosterone plus Unilateral Nephrectomy*. Studies have confirmed that after mice underwent unilateral nephrectomy and continued infusion of aldosterone (0.15 *μ*g/h) via an osmotic minipump together with 1% NaCl in drinking water for 4 weeks, myocardial fibrosis could be observed, and the survival rate was 100% in the mice [137–139]. Another research showed that after unilateral nephrectomy rats received high dose of aldosterone (0.75 *μ*g/h) via implanted minipump for 4 weeks, perivascular fibrosis was first detected in the experimental group [140]. However, Matsubara et al. observed perivascular fibrosis in the sixth week by using the same method [141]. This time point difference may be related to the different species of experimental animals.

It is worth noting that aldosterone-induced myocardial fibrosis occurs only when sodium intake is increased; aldosterone alone did not significantly induce fibrosis [142]. This model can be used to study the mechanism of target organ damage caused by aldosterone [143].

#### 3.6.4. L-NAME

Apart from aldosterone and angiotensin, scientists have considered that the NOS inhibitor, L-NAME, could be an important conception to induce myocardial fibrosis [144]. L-NAME causes vascular dysfunction by attenuating the vasodilating effects of nitric oxide, activates the RAAS system, and ultimately mediates elevated blood pressure [145]. Following L-NAME treatment, morphological changes in the rat heart mainly include ventricular hypertrophy, fibrosis, and necrosis [146].

Study has shown that, after 8 weeks of treatment with L-NAME (20 mg/d), 80% of the animals showed several areas of repairing fibrosis, and 33% of the rats died in the L-NAME group [147]. After feeding or gavage with L-NAME at 40 mg/kg/d for 4 weeks, the heart weight increased, cardiac collagen accumulation and interstitial fibrosis increased, and aortic section showed significant vascular fibrosis [148]. Another study showed that, after L-NAME (60 mg/kg/day) gavage to rats for 6, 12, and 16 weeks, interstitial and perivascular fibrosis was increased in a time-dependent way, and the mortality in rats treated with L-NAME was 10% at 16 weeks [149]. Research demonstrated that fibrosis around the coronary microvasculature increased after 6 weeks of treatment with L-NAME (3 mg/mL) in rats [150]. Low doses of L-NAME, 0.1 g/L through drinking water, applied for 14 days, also increased the blood pressure, cardiomyocyte cross-sectional area, and myocardial interstitial fibrosis [151]. What is more, Pechánová et al. confirmed that myocardial fibrosis was more pronounced at higher doses of L-NAME [152].

The model is characterized by the stable increase of blood pressure and simple replication method [153]. A recent study has shown that elevated blood pressure caused by L-NAME may be affected by diet [154].

### 3.7. Genetic Model

Genetic models may be obtained via gene deletion, overexpression, or mutation. A research showed that the cardiac function of Ufl1 (ubiquitin-fold modifier 1) knockout mice began to deteriorate at 2 months and significant cardiac chamber dilation with interstitial fibrosis appeared at 6 months [155]. Another research confirmed that the EC-specific Raf1^L613V^ expression mice had myocardial hypertrophy and myocardial fibrosis 4 days after birth [156].

This model is used to study gene expression and protein transcription during myocardial fibrosis, so that the potential biomarkers and therapeutic targets could be discovered [157]. The technique of targeting vector of genomic DNA fragment plays an important role in model construction [155, 156].

### 3.8. Others

There are some other methods to induce myocardial fibrosis, such as aortocaval fistula [158] and percutaneous artificial aortic stenosis [159]. Both of these methods can induce myocardial fibrosis, and both are used for disease mechanism research.

## 4. Discussion

A large number of studies in recent years have shown that myocardial fibrosis is closely related to many heart diseases. As a common pathological result of various heart diseases, its development mechanism has received extensive attention. However, myocardial fibrosis of humans characterized by disordered collagen arrangement cannot be completely simulated by any existing model because of its complex multifactorial pathogenesis. Now, there are both advantages and disadvantages in different animal models; hence, there is no uniform standard for the establishment of animal models of myocardial fibrosis (Table 1). When selecting animal models, researchers should consider not only their advantages and disadvantages but also the purpose and method of the experiment. For example, if you tend to study the genetic determinants of hypertension progression and pathophysiological changes, the SHR model is a good choice. Aortic constriction model is often selected to research myocardial fibrosis from the perspective of increased circulation resistance. Renal vascular model is suitable for demonstrating the mechanism of renin and sympathetic nerve system on target organ injury in hypertension. Pulmonary hypertension model applies to investigating the endogenous mechanism of right ventricular remodeling caused by pulmonary artery injury. Moreover, exogenous induction model is associated with ventricular remodeling caused by activation of the renin-angiotensin-aldosterone system. Gene models are more suitable for detecting changes in genes and proteins during myocardial fibrosis. Of course, the ultimate choice of the model needs to be considered comprehensively. The proportion of success/failure cases is an important evaluation indicator of the model; regretfully, it is less mentioned in the experiments. If this indicator can be supplemented, the experimental data will be more complete. With the advancement of technology, the method of establishing the model needs further improvement.

## Figures and Tables

**Table 1:** Methods to establish an animal model.

Model	Modeling method		Molding time	Mortality	Success rate
SHR	—		18 m [5, 9, 10]	—	>50% [9]

*Aortic Stenosis Model*					
TAC	Ligation of the aortic arch	18-G needle and 4-0 silk [15]	8 w	7.14%	85.71
		17-G needle and 6-0 silk [16]	8 w	—	—
		27-G needle and 7-0 silk	4 w [17–19] 1 w [20, 21]	<25% [22–25] 25%–50% [26–28] 50%–75% [29–31]	28% [32]
		20-G needle and 3-0 silk [33]	6 w	85%	—
		22-G needle and 4-0 silk [34]	2 w	—	—
		18-G needle and 5-0 nylon [35]	8 w	—	75.76%
		The titanium clamp (internal diameter: 0.45 mm) [36]	3 w	20%	—
		26 G clip [37], 30 G clip [38, 39]	4 w	—	—
Ascending aorta constriction model	Ligation of the ascending aorta	0.8 mm blunt steel wire and 3-0 silk [46]	6 w	7.7%	82.82%
		27-G needle and 7–0 silk [47]	8 w	70%	—
		A titanium clip (50%–60% of original diameter) [43]	21 m [48]	20% [43]	100% [48]
		O-rings (ID of 0.61 mm or 0.66 mm) [49]	4 w or 6 w	1.3%	—
AAC	Ligation of the abdominal aorta	22-G needle and 2-0 silk [52] and 22-G needle and 4-0 silk [53]	12 w	—	—
		24-G needle and 2-0 silk [54]	3 w	8%	—
		7-G needle and 4-0 silk [55]	4 w	18.8%	100%
		21-G needle and 4-0 silk [56]	2 w	—	—

*Renovascular model*					
2K1C	Ligation of unilateral renal artery	A polytetrafluoroethylene tubing (0.2 mm internal space) [59]	3 d	1.2%	73.3 ± 15.4%
		Stainless steel wire (diameter: 0.3 mm) [60]	3 w	—	100%
		A silver clip (0.2 mm internal space)	4 W [61], 15 d [62], 6 w [63], 7 w [64]	—	—
2K2C	Ligation of bilateral renal arteries	Two silver clips (0.3 mm internal space)	10 w [66, 67], 4 w [68]	—	—
1K1C	Ligation of unilateral renal artery after nephrectomy	A silver clip (0.15 mm internal gap)	4 w [70, 71]	—	—

*Unilateral nephrectomy model*		Five-sixth nephrectomy	8 w [73, 76], 5 w [78]	—	—
			4 w [77]	20%	—
		Right nephrectomy	10 d [74]	—	—

*Pulmonary hypertension model*					
PAB	Ligation of the pulmonary artery	A surgical clip (internal diameter of 0.35 mm)	3 w [83], 7 d [84]	—	—
		18-G needle and 7-0 prolene suture [85]	4 w	35.71%	—
		Vascular cuff (5 mm width) [86]	3 w	3.8%	—
		16-G or 18-G and 7-0 prolene suture [87]	21 d	—	—
		Semiclosed clip (outer size: 18-G needle) [85]	4 w	46.43%	—
MCT	Injection	60 mg/Kg	2 w [94], 3 w [93]	—	—
			4 w	31.58% [86] or 37.5% [95]	—
			35 d [96]	55%	—
		50 mg/Kg	2 w [97]	—	—
SU5416	Injection	20 mg/kg, 10% O_2_	5 w	12.5% [105] or 25% [108]	—
			8 w [106], 9 w [107]	—	—

*Exogenous induction model*					
NaCl	Feeding	8% NaCl	13 w [112]	68%	—
			6 w [114]	—	—
			8 w [115]	—	—
			4 w [113]	—	—
			14 w	67% [116] or 25% [117]	—
			18 w [118]	25%	—
		200 ng/kg/min [122], 1.5 µg/g/day [123], 0.83 *μ*g/kg/min [124], 400 ng/kg/min [125], 0.7 mg/kg/d [126]	4 w	—	—
Angiotensin II	Osmotic minipump	0.2 or 2.0 *μ*g/kg/min [121]	4 w	100%	—
		1.5 mg/kg/day [130], 1000 ng/kg/min [128], 1.1 mg/kg/day [129], 800 ng/min/kg [127]	2 w	—	—
Aldosterone alone	Osmotic minipump	0.2 mg/kg/day [135], 200 mg/kg/day [136]	4 w	—	—
		0.15 *μ*g/h [137–139]	4 w	100%	—
Aldosterone plus unilateral nephrectomy	Osmotic minipump	0.75 *μ*g/h [140]	4 w [140] or 6 w [141]	—	—
L-NAME	Feeding or gavage	20 mg/d [147]	8 w	33%	80%
		40 mg/kg [148]	4 w	—	—
		60 mg/kg/day [149]	16 w	10%	—
		3 m/ml [150]	6 w	—	—
		0.1 g/L [151]	2 w	—	—

*Genetic model*					
Ufl1 knockout mice			6 m [155]	—	—
Raf1L613V expression mice			4 d [156]	—	—

## Abbreviations

SHR: Spontaneously hypertensive rat
RASS: Renin-angiotensin-aldosterone system
TAC: Thoracic aortic constriction
AAC: Abdominal aortic coarctation
2K1C: Two-kidney one-clip
2K2C: Two-kidney two-clip
1K1C: One-kidney one-clip
PAB: Pulmonary artery banding
MCT: Monocrotaline
Ang II: Angiotensin II
ALD: Aldosterone.
